# Studies of the Interaction between Isoimperatorin and Human Serum Albumin by Multispectroscopic Method: Identification of Possible Binding Site of the Compound Using Esterase Activity of the Protein

**DOI:** 10.1155/2013/305081

**Published:** 2013-11-10

**Authors:** Samira Ranjbar, Yalda Shokoohinia, Sirous Ghobadi, Nooshin Bijari, Saeed Gholamzadeh, Nastaran Moradi, Mohammad Reza Ashrafi-Kooshk, Abbas Aghaei, Reza Khodarahmi

**Affiliations:** ^1^Nano Drug Delivery Research Center, Kermanshah University of Medical Sciences, Kermanshah 6734667149, Iran; ^2^Novel Drug Delivery Research Center, School of Pharmacy, Kermanshah University of Medical Sciences, Kermanshah, Iran; ^3^Department of Pharmacognosy and Biotechnology, Faculty of Pharmacy, Kermanshah University of Medical Sciences, Kermanshah 6734667149, Iran; ^4^Department of Biology, Faculty of Science, Razi University, Kermanshah, Iran; ^5^Isfahan Pharmaceutical Sciences Research Center, School of Pharmacy and Pharmaceutical Sciences, Isfahan University of Medical Sciences, Isfahan, Iran; ^6^Department of Epidemiology, Kermanshah University of Medical Sciences, Kermanshah, Iran

## Abstract

Isoimperatorin is one of the main components of *Prangos ferulacea* as a linear furanocoumarin and used as anti-inflammatory, analgesic, antispasmodic, and anticancer drug. Human serum albumin (HSA) is a principal extracellular protein with a high concentration in blood plasma and carrier for many drugs to different molecular targets. Since the carrying of drug by HSA may affect on its structure and action, we decided to investigate the interaction between HSA and isoimperatorin using fluorescence and UV spectroscopy. Fluorescence data indicated that isoimperatorin quenches the intrinsic fluorescence of the HSA via a static mechanism and hydrophobic interaction play the major role in the drug binding. The binding average distance between isoimperatorin and Trp 214 of HSA was estimated on the basis of the theory of Förster energy transfer. Decrease of protein surface hydrophobicity (PSH) was also documented upon isoimperatorin binding. Furthermore, the synchronous fluorescence spectra show that the microenvironment of the tryptophan residues does not have obvious changes. Site marker compettive and fluorescence experiments revealed that the binding of isoimperatorin to HSA occurred at or near site I. Finally, the binding details between isoimperatorin and HSA were further confirmed by molecular docking and esterase activity inhibition studies which revealed that drug was bound at subdomain IIA.

## 1. Introduction

Interaction between drugs and plasma proteins is an important pharmacological parameter which has a strong influence on their pharmacodynamic behaviors and affects the distribution and elimination of drug as well as the structure and physiological action of carrier proteins [1, 2]. However, problems associated with adsorption, distribution, metabolism, and elimination (ADME) add considerably to the complexity and cost of the development of new drugs and are driving the search for techniques to optimise these characteristics at an early stage in the design process. One of the most important factors affecting the distribution and the free, active concentration of many administered drugs is binding affinity for human serum albumin (HSA). HSA is the most abundant drug carrier protein, which is also found in tissues and bodily secretions throughout the body; the extravascular protein comprises 60% of the total albumin. HSA is a widely studied protein because its primary structure is well known and its tertiary structure has been determined by X-ray crystallography [3–6]. It has an important role in maintaining the colloidal osmotic pressure in blood; it transports and distributes exogenous and endogenous molecules and metabolites such as nutrients, hormones, fatty acids, and many diverse drugs [6, 7]. This heart-shaped protein which has a net charge of −15, at neutral pH, and an isoelectric point of about 5 consists of 585 amino acid residues and is composed of three structurally similar domains (I, II, and III), each containing two subdomains (A and B) and being stabilized by 17 disulphide bridges [4–8]. Crystal structure analyses have revealed that HSA has binding sites for aromatic and heterocyclic ligands within two hydrophobic pockets; in subdomains IIA (commonly referred to as Sudlow's site I: warfarin-binding site) and IIIA (commonly referred to as Sudlow's site II: indole/benzodiazepine site). Both hydrophobic and electrostatic interactions play a major role in controlling the affinity towards drug binding for sites I and II; for site I, mainly hydrophobic interactions are dominant, whereas for site II, a combination of hydrophobic, hydrogen bonding, and electrostatic interactions all play a crucial role. The sole tryptophan residue (Trp 214) of HSA is in subdomain IIA (site I) [6–8]. 

**Figure 1:**
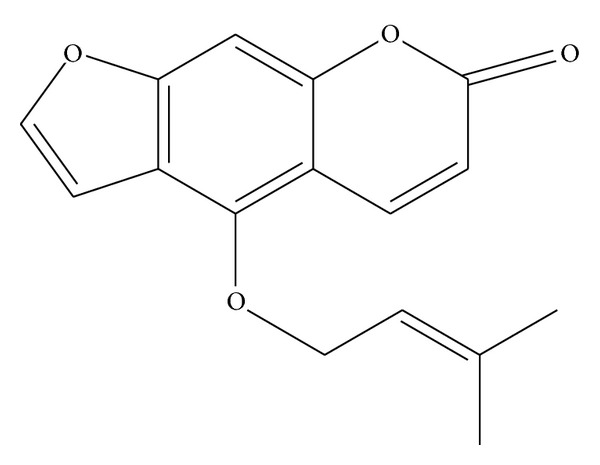
Structure of isoimperatorin.

Coumarins are widely distributed in nature and are found in all parts of plants. These compounds are especially common in grasses, orchids, citrus fruits, and legumes [9]. Being so abundant in nature, coumarins make up an important part of the human diet. Based on chemical structure, they can be broadly classified as (a) simple coumarins, (b) furanocoumarins of the linear or angular type, and (c) pyranocoumarins of the linear or angular type [9–11]. Isoimperatorin (4-[(3-methyl-2-butenyl) oxy]-7H-furo [3,2-g] [1] benzopyran-7-one) [12] (Figure 1) [13] is one of the main components of *Prangos ferulacea *as a linear furanocoumarin [14]. The plant has previously shown antispasmodic effects [15, 16] and proved to contain coumarins and terpenoids [17–19]. Isoimperatorin has attracted increasing interest due to its anti-inflammatory, analgesic, antispasmodic, and anticancer activities and treatment of Alzheimer's disease [9, 10, 20]. With the increasing importance of isoimperatorin in human health, it is necessary to evaluate its effect on biomolecules, for example, proteins, as a chemical entity [10, 20]. Interaction between serum albumin and many furocoumarins has been investigated successfully, but detailed investigations of the interaction of HSA with isoimperatorin are not available. So in this study we investigated the effect of isoimperatorin on the structure and action of HSA by means of various spectroscopic techniques. The UV-vis spectroscopy and intrinsic and extrinsic fluorometry methods were used for elucidating the mode of binding and probable structural alterations of HSA upon drug binding. The characteristics of the binding, that is, binding constant, number of binding sites, and and nature of binding forces, as well as the effects of the complexation on the protein conformation were determined. In addition, the thermodynamic and Förster's parameters associated with the binding process were also calculated. 

## 2. Materials and Methods

### 2.1. Chemicals

The pure warfarin was obtained as a generous gift from Dr. S. Kashanian (Razi University). 1-Anilinonaphthalene-8-sulfonate (ANS) was purchased from Sigma Chemical Co. (St. Louis, Mo, USA). The other reagents were of analytical grade from Merck (Darmstadt, Germany). All of the solutions were prepared in double-distilled water, and all of the experiments were carried out in 50 mM phosphate, pH 7.5, as the buffer. All of the measurements were done in triplicate.

### 2.2. Extraction, Isolation, and Identification of Isoimperatorin

Roots of *Prangos ferulacea* L. were collected from Yasuj in Kohgiluyeh-Boyer-Ahmad province, Iran, in June 2011. The plant was identified at the Botany Department of Yasuj University, Yasuj, and compared to the voucher specimen (No. 2408) at the Herbarium of School of Pharmacy, Isfahan University of Medical Sciences, Isfahan, Iran. 

Dried ground roots were extracted with acetone for two days (5 L × 4). The extract was concentrated to bear a viscous mass which was then put in −20°C for two days and chill filtered, in which the filtrate resulted in a solid mass after drying. The latter was fractioned by vacuum liquid chromatography (Silica gel, MESH 230–400) using a gradient of heptane : ethylacetate (H : EtOAc) from 100 : 0 to 0 : 100 to afford several fractions. Third fraction (H : EtOAc, 7 : 3) rendered a mixture of two coumarins in which further purification via chromatographies (H : EtOAc, 8 : 2 to 7 : 3) resulted in pure isoimperatorin. 

### 2.3. UV-vis Measurements

The UV-vis spectra are recorded at room temperature on a Cary Eclips (Varian) spectrophotometer equipped with 1.0 cm quartzecells. The final drug concentrations and constant protein concentration were 0.4–8 *μ*M and 3 *μ*M, respectively. The exact concentration of HSA was determined spectrophotometrically using molar extinction coefficient of 35700 M^−1 ^cm^−1^ [21].

### 2.4. Intrinsic Fluorescence Measurements

Fluorescence measurements were performed on a Cary Eclipse (Varian) spectrophotometer with jacketed cell holders in which temperature was adjusted by an external thermostated water circulation. The excitation and emission wavelengths were set at 295 nm (to avoid the contribution from tyrosine residues [22]) and 300–570 nm, respectively, with the excitation and emission slit widths of 5 and 10 nm, respectively. Fluorescence emission spectra of the HSA were measured in the absence and presence of the various concentrations of isoimperatorin (0.2, 0.4, 0.6, 0.8, 1, 1.5, 2, 2.5, 3, 3.5, 4, 5, 6, 8, and 10 *μ*M). The final enzyme concentration was 3 *μ*M.

Synchronous fluorescence spectra of HSA with various concentrations of isoimperatorin were obtained (Δ*λ* = 15 nm and Δ*λ* = 60 nm) with the excitation and emission slit widths of 5 and 10 nm, respectively.

#### 2.4.1. The Inner Filter Effect

The inner filter effect can be a problem for any fluorescence measurement especially where an absorbing component is being titrated into the cuvette. During a fluorescence titration experiment at the wavelength of excitation or at the wavelengths used to follow an emission, the absorption of the molecule added resulted in a spurious decrease in the observed fluorescence intensity [23]. In the steady-state fluorescence experiments, absorption spectrum of isoimperatorin overlaps with the excitation and emission of HSA. Thus, it is very important to subtract such an effect from the raw quenching data. The fluorescence data was corrected for inner filter effect using

(1) $$\begin{array}{c} F_{\text{corr}}=\;\frac{F_{\text{obs}}\;\text{antilog}\left(A_{\text{em}}+A_{\text{ex}}\right)}{2}, \end{array}$$

where *F*_corr_ and *F*_obs_ are the corrected and observed fluorescence intensity, *A*_ex_ and *A*_em_ are the optical density of the sample at the excitation and emission wavelengths, respectively.

#### 2.4.2. Determination of Quenching Mechanism

In order to clarify the mechanism of HSA fluorescence quenching by isoimperatorin, the quenching experiments were carried out at 298, 303, 308, and 313 K, where HSA does not undergo any considerable thermal denaturation. The decrease in fluorescence intensity at *λ*_max⁡_ was analyzed using the well-known Stern-Volmer equation [24]:

(2) $$\begin{array}{c} \frac{F_{0}}{F}=1+K_{\text{SV}}\left[Q\right], \end{array}$$

where *F*_0_ and *F* are the fluorescence intensities in the absence and presence of quencher (isoimperatorin), respectively, the *K*_SV_ is the Stern-Volmer quenching constant, and [*Q*] is the concentration of the compound isoimperatorin.

#### 2.4.3. Determination of Association Constant and Number of Binding Sites

The association constant (*K*_*b*_) and number of binding sites (*n*) for binding of small molecules to set of equivalent sites on a macromolecule can be determined using the modified Stern-Volmer equation:

(3) $$\begin{array}{c} \log\left\{\frac{F_{0}-F}{F}\right\}=\log K_{b}+n\log\left[Q\right], \end{array}$$

where *F*_0_ and *F* are the fluorescence intensities of HSA in the absence and presence of the quencher (isoimperatorin), respectively, *K*_*b*_ is the association constant, and *n* is the number of binding sites per HSA [25]. The values of *n* and *K*_*b*_ were obtained from the slope and intercept of the modified Stern-Volmer plot, respectively.

#### 2.4.4. Thermodynamic Analysis of the Binding Process

In order to explain the nature of the interaction between isoimperatorin and HSA, the association constant of HSA-isoimperatorin complex was determined at four different temperatures (298, 303, 308, and 313 K). The thermodynamic parameters of binding, entropy change (Δ*S*°) and enthalpy change (Δ*H*°), were obtained from van't Hoff equation:

(4) $$\begin{array}{c} \ln\;K_{b}=\frac{-\Delta H^{{}^{\circ}}}{RT}+\frac{\Delta S^{{}^{\circ}}}{R}, \end{array}$$

where *K*_*b*_ is the association constant at the given temperature and *R* is the universal gas constant. The values of Δ*S*° and Δ*H*° can be obtained from the intercept and slope of the van't Hoff plot, respectively, and the free energy change (Δ*G*°) can be estimated by using the Gibbs equation [26]:

(5) $$\begin{array}{c} \Delta G^{{}^{\circ}}=\Delta H^{{}^{\circ}}-T\Delta S^{{}^{\circ}}. \end{array}$$

### 2.5. Energy Transfer between HSA and Isoimperatorin

The distance between the acceptor (ligand) and the donor (tryptophan residues in the protein), *r*, according to Förster's nonradiation energy transfer theory, could be calculated by the following equation [21]:

(6) $$\begin{array}{c} E=1-\left(\frac{F}{F_{0}}\right)=\frac{R_{0}^{6}}{R_{0}^{6}+r_{0}^{6}}, \end{array}$$

where *E* is the efficiency of energy transfer between donor and acceptor, *F* and *F*_0_ are the fluorescence intensities of donor in the presence and absence of acceptor, respectively and *R*_0_ is a critical distance, at which the efficiency of energy transfer is 50%. The value of *R*_0_ was calculated using

(7) $$\begin{array}{c} R_{0}^{6}=8.8\times 10^{-25}K^{2}N^{-4}\Phi J, \end{array}$$

where *K* ^2^ is the spatial orientation factor describing the geometry of the donor and acceptor dipoles, *N* is the average refractive index of the medium, Φ is the fluorescence quantum yield of the donor in the absence of acceptor, and *J* expresses the degree of spectral overlap between the emission spectrum of the donor and the absorption spectrum of the acceptor. *J* can be given by

(8) $$\begin{array}{c} J=\sum F\left(\lambda \right)\varepsilon \left(\lambda \right)\lambda^{4}\Delta \lambda , \end{array}$$

where *F*(*λ*) is the fluorescence intensity of the donor at wavelength *λ* and *ε*(*λ*) is the molar absorption coefficient of the acceptor at *λ*. So *J* can be evaluated by integrating the overlap between emission spectrum of the donor and absorption spectrum of the acceptor [27].

The absorption spectrum of isoimperatorin (10 *μ*M) was recorded in the range of 222–450 nm. The emission spectrum of HSA (10 *μ*M) was also recorded in the range of 222–450 nm with excitation and emission slit widths of 5 and 5 nm, respectively. The overlap of the absorption spectrum of isoimperatorin with the fluorescence emission spectrum of HSA was used to calculate the efficiency of energy transfer. 

### 2.6. Protein Surface Hydrophobicity (PSH) Determination

The ANS binding properties of HSA can be investigated by measuring *K*_*d*_^app^ and *F*_max⁡_ of the HSA-ANS complex, in the absence and presence of isoimperatorin. *K*_*d*_^app^ is the apparent dissociation constant of the fluorescent ANS-HSA complex, and 1/*K*_*d*_^app^ is the binding affinity of ANS to the protein in the absence and presence of isoimperatorin [28]. *F*_max⁡_ is the maximum fluorescence intensity at the saturated ANS concentration which indicates the number of surface hydrophobic sites of the protein. This method involves titration of the enzyme solutions in the presence of increasing concentrations of ANS. The values of 1/*K*_*d*_^app^ and *F*_max⁡_ can be obtained from the slope and the *x*-intercept of the Scatchard plot, respectively. In this type of Scatchard plot, the fluorescence intensity/[ANS]_free_ is drawn versus fluorescence intensity. The free ANS concentration must be determined via plotting fluorescence intensity versus total ANS concentration. In dilute solutions of ANS, a linear relationship exists between fluorescence intensity (*F*) and ANS concentration (*c*) (*F* = *Bc*, where *B* is the proportionality coefficient between fluorescence intensity and ANS concentration). From a 1 mM stock solution, ANS was added to the final concentrations ranging from 0 to 50 *μ*M. The enzyme concentration was 1 *μ*M. It is assumed that, in very dilute ANS solutions (0-1 *μ*M), all of the ANS molecules are bound to the HSA, and thus, B (the proportionality coefficient between fluorescence intensity and ANS concentration) can be determined from the slope of the linear portion of fluorescence intensity plot versus ANS concentration. For each of the different total concentrations of ANS used, the concentration of bound ANS molecules was calculated using the following equation: [ANS]_bound_ = *F*/*B*, and the free ANS molecules concentration was calculated from the difference between total and bound ANS concentrations ([ANS]_free_ = [ANS]_total_ − [ANS]_bound_) [29, 30].

The protein surface hydrophobicity index in the absence and presence of the drug can be calculated by applying the following equation [28]:

(9) $$\begin{array}{c} \text{PSH}=\frac{F_{\max}}{\left[\text{HSA}\right]K_{d}^{\text{app}}}. \end{array}$$

Following excitation at 380 nm, the fluorescence emission increment was recorded at 470 nm until no further increase in fluorescence intensity was observed.

### 2.7. Competitive Experiments

In order to elucidate the binding site of isoimperatorin on HSA, we carried out competitive experiments at the presence of two known drugs (Warfarin, as site I marker, and Ibuprofen, as a drug that preferentially binds to site II) [24, 31]. The concentration of HSA and warfarin/ibuprofen was stabilized at 1.0 × 10^−6^ M. Isoimperatorin was then gradually added to the HSA, HSA-warfarin or HSA-ibuprofen mixtures. An excitation wavelength of 295 nm was selected, and flourescence spectra were recorded in the range of 300–450 nm. 

Also, possible inhibitory effect of isoimperatorin, indomethacin, tetracycline, ibuprofen, aspirin, osthole and warfarin on the esterase activity of HSA was evaluated.

### 2.8. Docking Procedure

The AutoDock 4.0.5 docking program was applied to study the interaction between the isoimperatorin and HSA. The crystal structure of HSA was obtained from the Protein Data Bank. Of more than 50 X-ray crystallographic structures related to human serum albumin, in Protein Data Bank [32], entry with PDB ID:1BM0 [33] was chosen for dockings because of no missing atoms, no cocrystallized ligand, and having a reasonably good resolution (2.5 Å). All the water molecules were removed using a plain text editor. The polar hydrogen, partial charge, and solvation parameters for use with the AutoDock 4.0.5 simulation were applied using AutoDock-Tools. The docking simulations were carried out with the rigid HSA and a flexible ligand [34]. Using AutoGrid tools, the grid maps were generated adequately large to include the binding site(s) of protein as well as significant regions of the surrounding surface. The points of the grids were thus 126 × 126 × 126 with a grid spacing of 0.375 Å [35]. Afterwards, the most suitable structure for the flexible ligand molecule was optimized by the rotation of all single bonds in the ligand molecule. The grid parameter file and the docking parameter file were set up by the AutoDockTools program [36]. The binding mode of isoimperatorin was predicted by using the Lamarckian genetic algorithm search engine implemented in AutoDock software package [37] for the two main drug binding sites I and II (subdomains IIA and IIIA of HSA, resp.) [38]. Population size was 256, and Cluster analysis was performed on the docked results using a root mean square (RMS) tolerance of 0.5 Å. Default settings were used for all other parameters [35]. At the end of the docking simulation, the HSA-IIM complex with the most stable energy was adopted as the most favorable structure of the complex for HSA. Molecular graphics were prepared with PyMOL version 0.99 beta06 (http://www.pymol.org/).

## 3. Results and Discussion

### 3.1. UV Absorption Spectra

**Figure 2:**
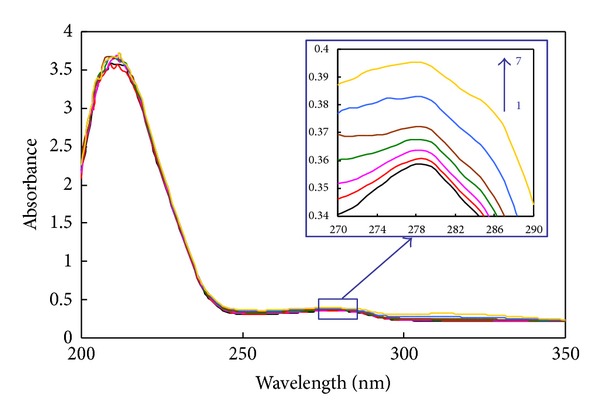
Absorption spectra of HSA (3.0 *μ*M) with various amounts of isoimperatorin: (1–7) 0, 0.4, 0.8, 2, 4, 6, and 8 *μ*M. The absorption of each sample was measured with respect to the respective blank.

UV absorption spectroscopy technique can be used to explore the structural changes of protein and to investigate protein-ligand complex formation. HSA has two main absorption bands that one of them is located in the range of 260–300 nm which is the absorption band of the aromatic amino acids (Trp, Tyr, and Phe) [27, 39].  Figure 2 shows the UV absorption spectra of HSA in the absence and presence of isoimperatorin. As can be seen in Figure 2, HSA has strong absorbance with a peak at 280 nm and the absorbance of HSA increased with the addition of isoimperatorin. In addition, the absorbance value of 280 nm of the isoimperatorin at a concentration of 10 *μ*M is very low (<0.03). Our results confirmed that the interaction between HSA and ligand took place.

### 3.2. Fluorescence Measurements

#### 3.2.1. Intrinsic Fluorescence Measurements

**Figure 3:**
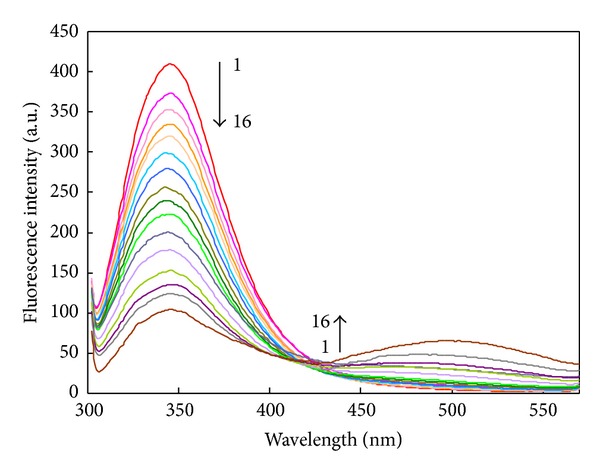
Effect of isoimperatorin on fluorescence spectra of HSA (*T* = 298 K, pH: 7.5). Curve (1): 3.0 *μ*M HSA; Curves (2–16): 3.0 *μ*M HSA in the presence of 0.2, 0.4, 0.6, 0.8, 1, 1.5, 2, 2.5, 3, 4, 5, 6, 7, 8, and 10 *μ*M of isoimperatorin.

The fluorescence spectra of HSA in the absence and presence of different amounts of Isoimperatorin were recorded in the range of 300–570 nm upon excitation at 295 nm. isoimperatorin causes a concentration dependent quenching of the intrinsic fluorescence of HSA (Figure 3) without changing the emission maximum and shape of the peaks. These results indicated that there were interactions between isoimperatorin and HSA. This interaction was further confirmed by UV-vis absorption technique.

A gradual decrease in the fluorescence emission intensity of HSA is observed in the presence of increasing concentrations of isoimperatorin suggesting the presence of tryptophan residue of the HSA at or near the isoimperatorin binding site. HSA has one Trp (Trp 214) and its fluorescence is solely due to the excitation of this residue of HSA located in the binding domain-IIA (site I) at the bottom of a 12 Å deep crevice [40].

Emerging of a new emission peak at 505 nm upon isoimperatorin addition can be attributed to the bound drug and radiationless energy transfer between the HSA tryptophan, and isoimperatorin [41]. The appearance of an isoactinic point at 430 nm might also indicate the existence of equilibrium between bound and free drug. In addition, such equilibrium may emphasize the formation of the drug-protein complex [42].

#### 3.2.2. Fluorescence Quenching Mechanism

Quenching can occur by different mechanisms, which is usually classified as dynamic quenching and static quenching. In dynamic quenching, increasing the temperature results in faster diffusion and hence larger amounts of collision which causes raising of the quenching constant. Opposing to dynamic quenching, in static one, increasing the temperature weakens the stability of the formed complex and hence reduces the quenching constant [19].

**Figure 4:**
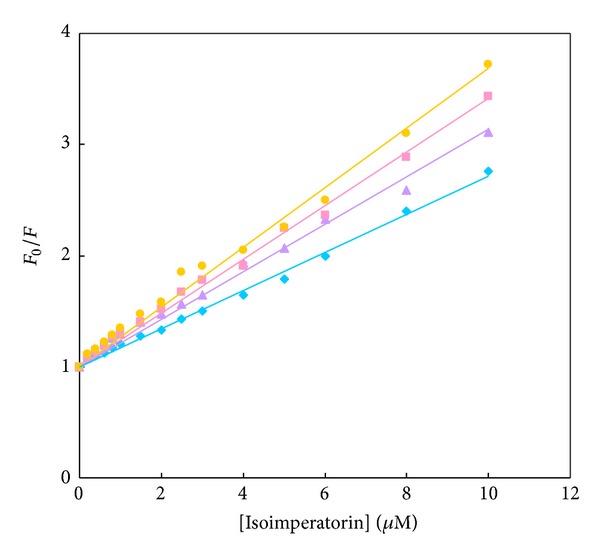
The Stern-Volmer plots for HSA fluorescence quenching by isoimperatorin at 298 (*◆*), 303 (▲), 308 (■), and 313 (●) K. The final protein concentration in 50 mM phosphate, pH 7.5, was 3 *μ*M. Based on “One-Sample Kolmogorov-Smirnov Test,” test distribution was normal.

Fluorescence quenching of HSA upon interaction with isoimperatorin was evaluated at different temperatures (298, 303, 308, and 313 K), and the results are shown in Figure 4. As indicated in this figure, the Stern-Volmer plots are linear with the slopes increasing with increasing temperature (the linearity in this plot confirmed one-to-one interaction between isoimperatorin and HSA). The calculated values of *K*_SV_ at different temperatures are listed in Table 1. It can be found from Table 1 that the value of *K*_SV_ increased with increasing temperature in the presence of isoimperatorin which indicates the involved binding forces are mainly hydrophobic interactions (endothermic apolar interactions are strengthened with increasing temperature). Also, the rate constant of the quenching process (*k*_*q*_) which is equal to *K*_SV_ divided by *τ*_0_ (with assuming *τ*_0_ = 10^−8^ s)

(10) $$\begin{array}{c} k_{q}=\frac{K_{\text{SV}}}{\tau_{0}} \end{array}$$

was calculated (Table 1). The results revealed that the values of *k*_*q*_ were much greater than the maximum diffusion rate constant of the biomolecule (2 × 10^10^ M^−1^ s^−1^), which indicated that the fluorescence quenching of HSA was initiated by complex formation between HSA and isoimperatorin, confirming that the mechanism of quenching is static [19]. 

#### 3.2.3. Determination of Association Constant (*K*_*b*_) and Number of Binding Sites (*n*)

**Figure 5:**
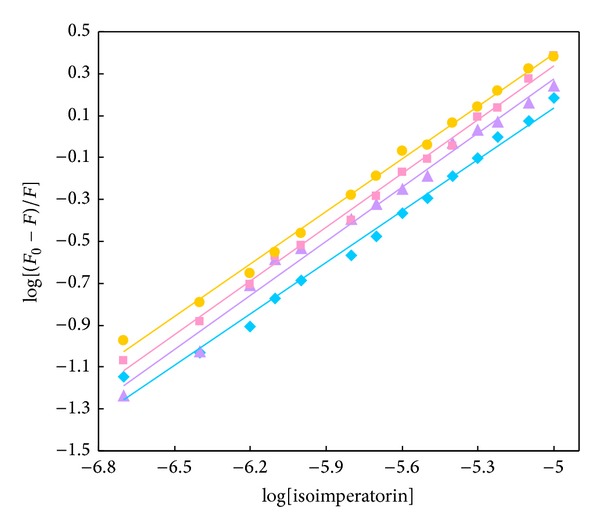
The modified Stern-Volmer plots for HSA fluorescence quenching by isoimperatorin at 298 (*◆*), 303 (▲), 308 (■), and 313 (●) K. For interpretation of references to color in this figure legend, the reader is referred to the web version of this article.

The values of association constant, *K*_*b*_, and the number of binding sites, *n*, at different temperatures, are obtained from the modified Stern-Volmer plots (Figure 5) and listed in Table 1. It can be found from these data that the *n* values at different temperatures are kept around unity, which shows the existence of one binding site in HSA for isoimperatorin. The value of *K*_*b*_ is significant to understand the distribution of the drug in plasma since the weak binding can lead to a short life time or poor distribution, while strong binding can decrease the concentration of free drug in plasma. The value of *K*_*b*_ illustrated that there is a strong binding force between isoimperatorin and HSA. It was found that the binding constant increased with increase in temperature resulting in an increment of the stability of the isoimperatorin-HSA complex; it also implies that isoimperatorin can be tightly stored and carried by HSA in the body.

#### 3.2.4. Forces Involved in the Binding Process

Considering the dependence of binding constant on temperature, a thermodynamic process was considered to be responsible for the formation of a complex. Therefore, the thermodynamic parameters dependent on temperatures were analyzed. The acting forces between a small molecule and macromolecule mainly include hydrogen bonds, van der Waals forces, electrostatic forces, and hydrophobic interaction forces [23]. In order to obtain the information about the forces persisting in the present binding process, the thermodynamic parameters of binding were obtained from Van't Hoff plot followed by the Gibbs equation (data not shown).  Table 2 shows the values of Δ*H*° and Δ*S*° obtained for the binding site from the slopes and ordinates at the origin of the fitted lines. From Table 2, it can be seen that the negative sign for free energy (Δ*G*°) means that the interaction process is spontaneous. The positive enthalpy (Δ*H*°) and entropy (Δ*S*°) values of the interaction of isoimperatorin and HSA indicate that the binding is mainly entropy-driven and the enthalpy is unfavorable for it; the hydrophobic forces play a major role in the reaction [43].

### 3.3. Nonradiative Energy Transfer from HSA to Isoimperatorin

The Förster nonradiative energy transfer (FRET) has been used as a “spectroscopic ruler” for measurement of the distance between the acceptor (ligand) and the donor (tryptophan residues in the protein) [44]. According to this theory, there will be FRET between two molecules if the following conditions were satisfied: (a) the donor molecule emits fluorescence; (b) the emission spectrum of the donor has an enough overlap with the absorption spectrum of the acceptor; and (c) the donor and acceptor molecules are close enough with the maximum distance not to exceeding 7 nm [45]. 

**Figure 6:**
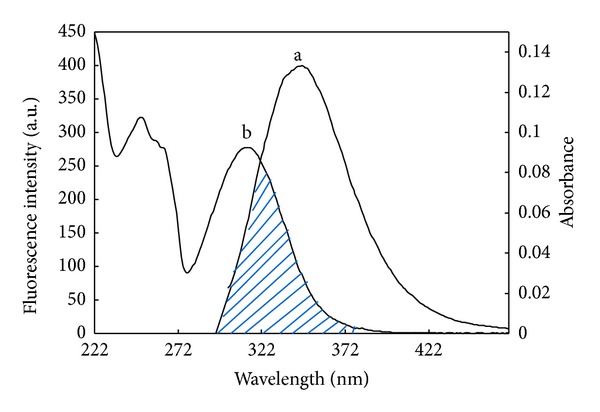
The overlap of the fluorescence spectrum of HSA (a) and the absorption spectrum of isoimperatorin (b) when [HSA] = [isoimperatorin] = 10 *μ*M. The enzyme was excited at 295 nm.

There is a good overlapping between the fluorescence emission spectrum of HSA and absorption spectra of isoimperatorin (Figure 6). As the fluorescence emission of protein was affected by the excitation light around 295 nm, the spectrum ranging from 300–400 nm was chosen to calculate the overlapping integral. The value of *J* was calculated to be 4.73 ± 0.05 × 10^−15^ cm^3^ L mol^−1^ by integrating the overlap spectra for 300–400 nm. In the present case, *K* ^2^ = 2/3, *N* = 1.36 and Φ = 0.118 [21], according to (6) and (7), we could calculate that *E* = 0.45 ± 0.06, *R*_0_ = 3.22 ± 0.3 nm, and *r* = 2.9 ± 0.2 nm. As mentioned earlier (Section 3.2.1), HSA posses one tryptophan residue (Trp 214) and the calculated *r* is actually the average value between the bound drug and Trp 214. Since *r* is much smaller than 7 nm and 0.5*R*_0_ < *r* < 1.5*R*_0_ [41], therefore the energy transfer from HSA to isoimperatorin occurs with high probability, which also explains the efficient quenching of the HSA fluorescence. 

### 3.4. PSH Determination in the Absence and Presence of Isoimperatorin

**Figure 7:**
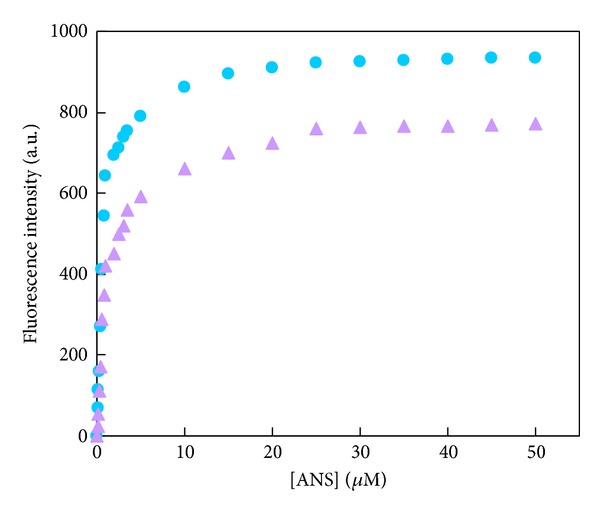
Binding of ANS to HSA (1 *μ*M) in the absence (●) and presence (▲) of 3 *μ*M of isoimperatorin.

Several methods exist for measuring the surface hydrophobicity of proteins that ANS experiment is one of them. ANS, as an extrinsic fluorescent probe, is extremely sensitive to polarity of the solvent. In aqueous solutions, it fluoresces very weakly, but upon binding to hydrophobic patches of proteins, its quantum yield increases significantly [29]. Thus, ANS fluorescence may be applied for monitoring possible changes in protein surface hydrophobicity induced upon drug binding. At a fixed concentration (1 *μ*M) of HSA and increasing concentrations of ANS (0–50 *μ*M), fluorescence intensity of ANS was measured in the absence and presence of 3 *μ*M isoimperatorin (Figure 7). 

**Figure 8:**
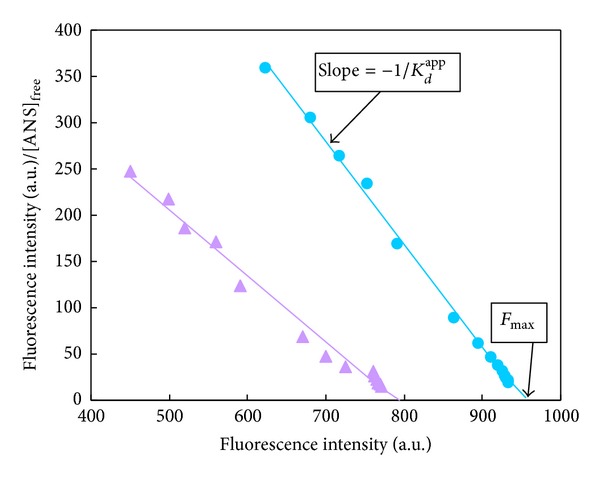
Scatchard plots for titration of HSA with increasing concentrations of ANS in the absence (●) and presence (▲) of 3 *μ*M of isoimperatorin. *F*_max⁡_ and 1/*K*_*d*_^app^ values were obtained from the *x*-intercept and slope of this plot. For interpretation of references to color in this figure legend, the reader is referred to the web version of this paper.

A typical hyperbolic response is observed which demonstrates the saturation nature of ANS binding to HSA. It can be seen that the hyperbolic response of ANS fluorescence in the presence of isoimperatorin is slightly different in comparison to the absence of the drug. The values of *F*_max⁡_ and *K*_*d*_^app^ for ANS are obtained from the Scatchard plot (Figure 8) and listed in Table 3. The value of *K*_*d*_^app^ for ANS binding in the absence of drug (0.9 ± 0.05) was increased in the presence of 3 *μ*M isoimperatorin (1.4 ± 0.07), which shows the weaker binding of ANS to the HSA-isoimperatorin complex. Then, PSH was calculated in the absence and the presence of 3 *μ*M isoimperatorin by using *F*_max⁡_ and *K*_*d*_^app^ values (Table 3). Decreasing the PSH index of HSA (from 1507.94 ± 11.46 to 806.12 ± 0.07) suggests that the surface hydrophobicity of HSA is decreased by ~46.5% upon drug binding. 

### 3.5. Synchronous Fluorescence Spectroscopy

**Figure 9:**
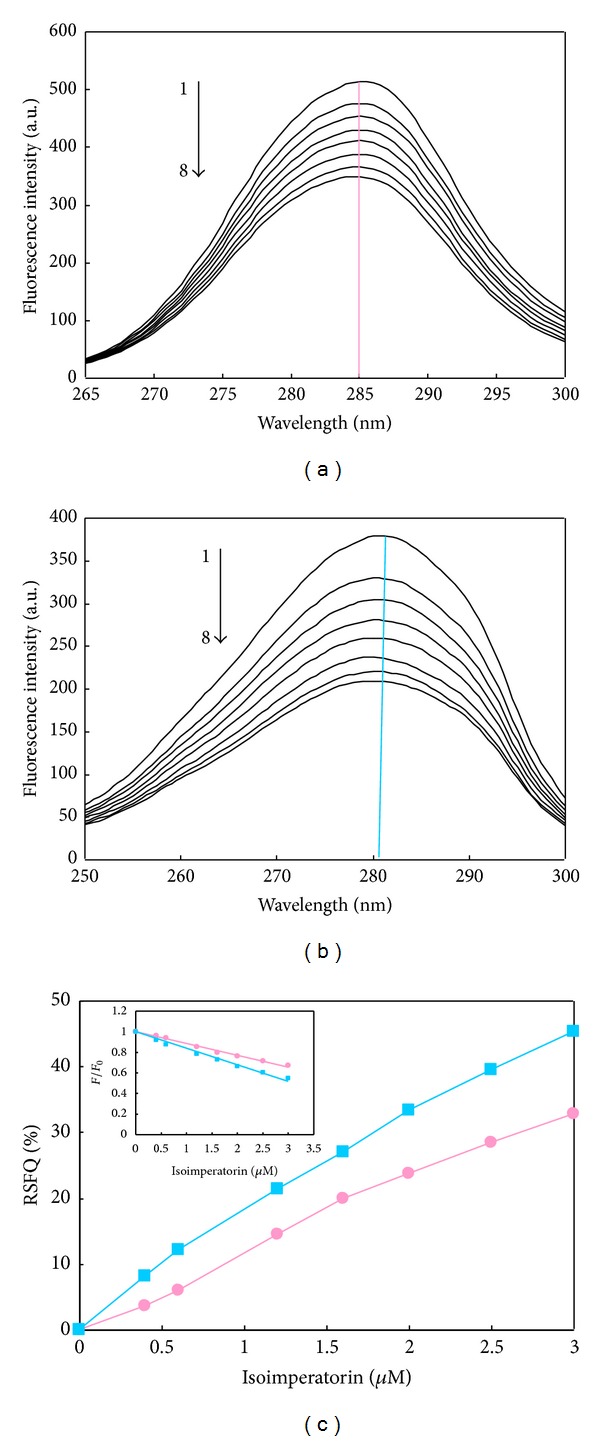
Synchronous fluorescence spectra of interaction between HSA and isoimperatorin (a) at Δ*λ* = 15 nm and (b) at Δ*λ* = 60 nm. Concentration of HSA was 3.0 *μ*M, while concentrations of isoimperatorin were 0, 0.4, 0.8, 1.2, 1.6, 2, 2.5, and 3 *μ*M from 1 to 8. (c) Comparative evaluation of isoimperatorin effect on the RSFQ of HSA; [HSA] = 3 *μ*M, (■) Δ*λ* = 15 nm, and (●) Δ*λ* = 60 nm.

Synchronous fluorescence spectroscopy introduced by Lody has been used to characterize complex mixtures, which can provide fingerprints of complex samples [46]. The synchronous fluorescence spectra can provide information about the molecular environment in the vicinity of the chromophore molecules. In the synchronous fluorescence spectra, the sensitivity associated with fluorescence is maintained while several advantages are available: spectral simplification, spectral bandwidth reduction and avoidance of different perturbing effects. The fluorescence spectrum of BSA/HSA mainly due to the Trp residues is sensitive to the microenvironment of these chromophores with the maximum emission wavelength being very useful in estimating the hydrophobicity around the tryptophan residues. It is well known that the experiment on synchronous fluorescence of HSA will provide the characteristic information for the Tyr and Trp residues when the scanning interval Δ*λ* is fixed at 15 and 60 nm, respectively [47]. The shift in the position of fluorescence emission maximum corresponds to changes of the polarity around the chromophore molecule. A blue shift of *λ*_max⁡_ means that the amino acid residues are located in a more hydrophobic environment and are less exposed to the solvent, while a red shift of *λ*_max⁡_ implies that the amino acid residues are in a polar environment and are more exposed to the solvent [48]. The synchronous fluorescence spectra of interaction between isoimperatorin and HSA are presented in Figure 8. It was shown that the fluorescence intensity of HSA decreased regularly along with the addition of isoimperatorin, which further demonstrated the occurrence of fluorescence quenching in the binding process. Moreover, there is no significant shift of the maximum emission wavelength with Δ*λ* = 15 nm or 60 nm (Figures 9(a) and 9(b)), which implies that interaction of isoimperatorin with HSA does not affect the conformation of the region around the Tyr or Trp residues. 

As well, we can obtain the information about the location of isoimperatorin binding site from the synchronous fluorescence data. The ratios of synchronous fluorescence quenching (RSFQ) were used to determine the binding sites of the drugs to the HSA molecule in drug-HSA complexes. The RSFQ values were calculated from the following equation:

(11) $$\begin{array}{c} \text{RSFQ}=1-\frac{F}{F_{0}}, \end{array}$$

where *F* and *F*_0_ are the synchronous fluorescence intensities of HSA in the presence and the absence of drug, respectively. It was obvious from Figure 9(c) that the RSFQ results for Δ*λ* = 60 nm were slightly higher than the corresponding ones for Δ*λ* = 15 nm. As illustrated in Figure 9(c), addition of isoimperatorin resulted in strong fluorescence quenching of Trp residue (~47% reduction in fluorescence intensity), confirming that isoimperatorin reached subdomain IIA, where only one Trp residue (Trp 214) in HSA is located. It was also observed from Figure 9(c) that the fluorescence strength of Tyr residues decreased about ~32% in the presence of isoimperatorin. So the binding of isoimperatorin to HSA quenched flourescence intensity of Tyr and Trp. Since Trp 214 (the only Trp of HSA) is at site I and Tyr 263 (has the strong fluorescence in the case of Tyr residues and may be one of the residues responsible for the synchronous fluorescence spectrum of HSA when Δ*λ* is fixed at 15 nm) is in subdomain IIA (site I), it can be concluded that Trp and Tyr residues of HSA may have equal accessibility to binding site of isoimperatorin [49]. The above results indicated that isoimperatorin can bind to HSA in the hydrophobic cavity (site I) on subdomain IIA, which are in full agreement with quenching, competitive binding experiments, and theoretical docking technique.

### 3.6. Site Marker Competitive Binding Experiments

**Figure 10:**
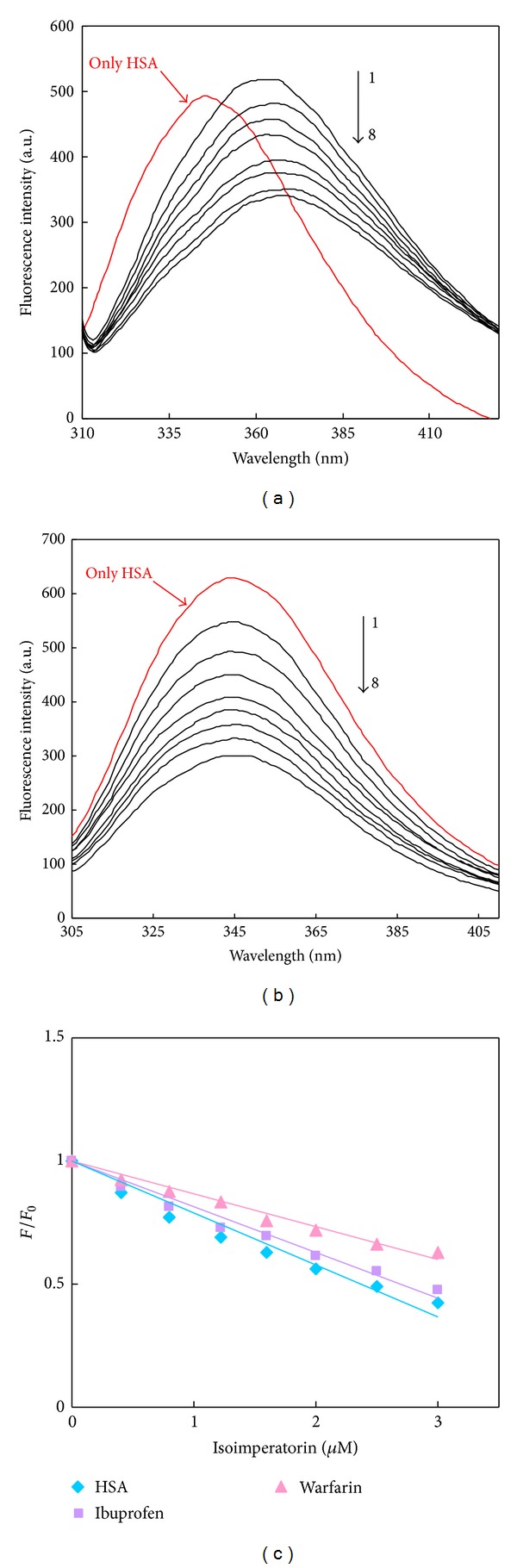
Effect of isoimperatorin on the quenching of HSA-warfarin complex (a) and HSA-ibuprofen complex (b) fluorescence. From 1 to 8: [HSA] = [warfarin or iboprufen] = 3 *μ*M, [isoimperatorin] = 0, 0.4, 0.8, 1.2, 1.6, 2, 2.5, and 3 *μ*M. All reproduced data shown are representative of at least 3 independent experiments. (c) Fluorescence quenching profiles of HSA (*◆*), HSA-ibobrofen (■), and HSA-warfarin (▲) in the presence of various concentration of isoimperatorin.

In order to further classify drug binding site on HSA, competitive binding experiments have been carried out. Warfarin and ibuprofen are two markers specific for binding to HSA that bind to sites I and II, which are corresponded to the subdomains IIA and IIIA, respectively. Therefore, to determine the location of the isoimperatorin binding site on HSA, the competitive displacement experiments were carried out using warfarin as a characteristic marker for site I (Figure 10(a)) and ibuprofen for site II (Figure 10(b)) [24, 31]. In the site marker competitive experiment, isoimperatorin was gradually added to the solution of HSA, HSA-ibuprofen or HSA-warfarin complex, and then, fluorescence intensity of system was recorded. 

**Figure 11:**
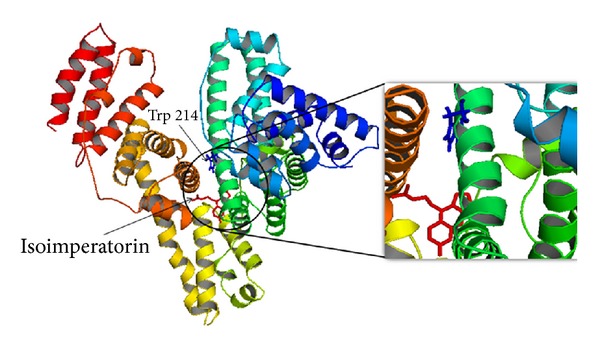
Crystal structure of HSA and the positions of single tryptophan residue (Trp 214) and also the binding site of isoimperatorin in HSA. Protein backbone is shown in the “cartoon” representation, and isoimperatorinand Trp 214 residue in the site I are shown in the “stick” representation. The isoimperatorin and Trp 214 structures are represented by the red and blue colors. The crystal structure of protein was obtained from Protein Data Bank (1BM0). For interpretation of references to color in this figure legend, the reader is referred to the web version of this paper.

As shown in Figure 10(a), with addition of warfarin in the HSA solution, the flourescence intensity was slightly higher than that of without warfarin (and including the red shift). Then, after adding the isoimperatorin into the above system, the flourescence intensity of HSA solution, with warfarin held in equimolar, decreased gradually, and the intensity was much lower than that of without warfarin, displaying that the binding of the isoimperatorin to HSA was affected after adding warfarin. On the contrary, in the presence of ibuprofen, the flourescence intensity of the HSA-ibuprofen complex almost had no difference from that recorded without ibuprofen under the same conditions (Figure 10(b)), which indicated that site II marker did not prevent the binding of isoimperatorin in its usual binding location. The next step, protein intrinsic fluorescence was measured, and the relative fluorescence intensities (*F*/*F*_0_) versus ligand concentration ([isoimperatorin]) plots are displayed in Figure 10(c). As shown in Table 4, the binding constant was remarkably decreased after addition of warfarin, while the addition of ibuprofen results in only a small difference. These results indicate that warfarin can displace the isoimperatorin, but ibuprofen has little effect on the binding of isoimperatorin to HSA. In other words, these results suggest that isoimperatorin probably competes with warfarin for binding to HSA [24]. Experimental observations were followed up with docking studies where isoimperatorin was docked to HSA to determine the preferred binding site on the protein. As described above, the 3D crystal structure of HSA is a monomer consisting of three homologous domains which assemble to form a heart-shaped molecule [4, 6]. Each of the structurally similar *α*-helix domains (I–III) has two subdomains (A and B), with six *α*-helices in subdomain A and four *α*-helices in subdomain B. The crystal structure analyses indicate that the principal regions of ligand binding sites in HSA are located in hydrophobic cavities in subdomains IIA and IIIA, which are consistent with Sudlow's site I and Sudlow's site II, respectively. Several studies have shown that HSA is able to bind many ligands in several binding sites [38]. In this paper, the molecular docking model of the HSA–isoimperatorin complex was conducted on the basis of the crystal structure of HSA to find the binding position of isoimperatorin. The best docking energy result is shown in Figure 11. It can be seen that isoimperatorin was situated within subdomain IIA in Sudlow's site I formed by six *α*-helices. This figure shows the location of Trp 214 and also the binding of isoimperatorin in HSA. As this fluorescent residue is in subdomain IIA, and due to the proximity of the binding site of isoimperatorin to the Trp 214, it can be concluded that isoimperatorin has one reactive site on HSA and probably this site is at or near the site I. The above docking results are in good agreement with the displacement experiments results and revealed that isoimperatorin binds to Sudlow's site I of HSA.

### 3.7. Effect of Isoimperatorin on HSA Esterase Activity

**Figure 12:**
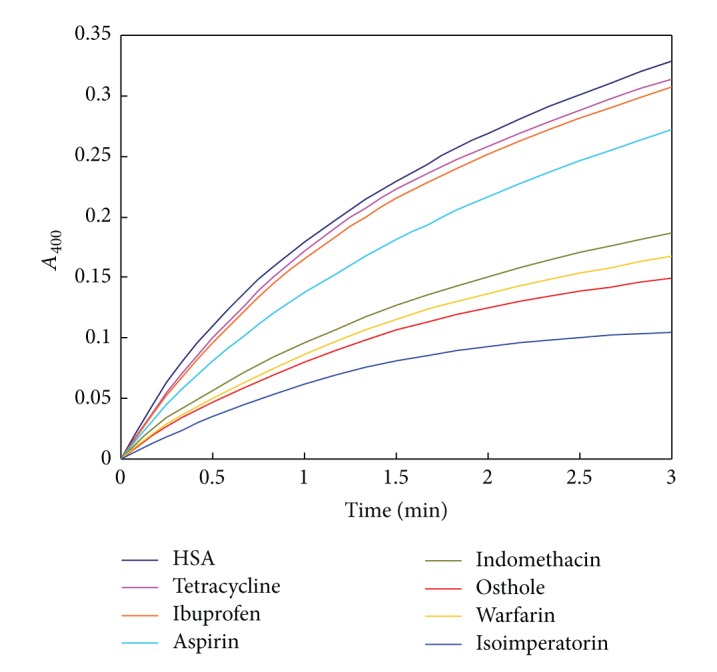
4-Nitrophenyl acetate esterase activity of HSA in the absence and presence of tetracycline and ibuprofen (site II), warfarin, aspirin, indomethacin, and osthole (site I), and isoimperatorin (site I). Final concentration of protein, pNPA, and drugs were 15, 250, and 250 *μ*M, respectively. For interpretation of references to color in this figure legend, the reader is referred to the web version of this paper.

Preliminary studies showed that albumin, in addition to its ligand binding capabilities, has remarkable promiscuous catalytic activities toward a broad range of organic molecules, including esters, amides, phosphates, and benzisoxazoles. Human serum albumin had significantly greater esterase-like activity than bovine serum albumin, and defatted albumin was more active than untreated albumin [50]. Interestingly, HSA exhibits esterase-like activity and hydrolyzes drugs having an ester group, such as aspirin (which can convert aspirin “acetylsalicylic acid” to salicylic acid). Lys199, Lys402, Lys519, and Lys545 form the pseudoesterase active site of HSA that is located in site I [50]. “Esterase-like” activity (hydrolysis of p-nitrophenol esters, such as p-NPA) by subdomain III A has been also reported, and site directed mutagenesis studies have shown that Arg 410 and Tyr 411 are essential in this esterase activity. However, further investigation revealed that acetylation is not limited to a distinct side chain so that the HSA incubation with high concentrations of pNPAc results in the acetylation of 59 Lys, 10 Ser, 8 Thr, 4 Tyr, and 1 Asp [51]. Because of the above difference, here, we set out to evaluate the effect of isoimperatorin on the esterase activity of HSA, based on our previous work [19]. The kinetics of the pseudoenzymatic hydrolysis of pNPAc was obtained in the absence and presence of isoimperatorin and site markers (Figure 12). Accordingly, warfarin and indomethacin (as site I marker) and isoimperatorin inhibited competitively the HSA-catalyzed hydrolysis of pNPAc by binding to site I. In contrast, ibuprofen and tetracycline were unable to inhibit esterase activity of HSA. The enzyme inhibition results suggested that isoimperatorin, aspirin, warfarin, indomethacin, and osthole binding sites on the HSA molecule are the same. These results also proposed that the large site I on HSA participates effectively to its promiscuous esterase activity.

## 4. Conclusion

The accumulated data on HSA-drug interactions can be used to develop quantitative structure-activity relationships for albumin binding. However, as stated earlier, adsorption, distribution, metabolism, and elimination (ADME) is controlled by albumin [30]. Poor or strong binding to SA is believed to affect half-time of drugs [35], and their dosage depends on their half-life in plasma. Thus, the binding affinity of any drug to SA is one of the major factors that determine the pharmacokinetics, halftime, and availability of the drug in various tissues (see [30] and references therein). Further complexities arise *in vivo* due to interactions between drugs and endogenous ligands for HSA. This is particularly pertinent for fatty acids, which normally occur in serum at levels between 0.1 and 2 mol per mol of HSA and can both compete and cooperate with drugs binding to the protein. In certain disease states, these effects are exacerbated as the fatty acid : HSA molar ratio may be as high as six. Other pathological conditions are associated with high (micromolar to millimolar) levels of bilirubin, hemin, or renal toxins (e.g., 3-carboxy-4-methyl-5-propyl-2-furanpropanoic acid (CMPF), indoxyl sulphate) which bind to the protein causing significant drug binding defects. 

Our major aim in this study was to distinguish the nature of the interaction of isoimperatorin to HSA using different biophysical techniques. This study could be very valuable due to importance and presence of isoimperatorin in human diet (food-drug interaction on HSA). The results showed that isoimperatorin quenched the intrinsic fluorescence of HSA through dynamic quenching mechanism. Furthermore, hydrophobic interaction played a major role in the binding process. Using Fluorescence and UV absorption spectroscopy, it has been shown that isoimperatorin induces protein structural changes. The binding average distance between the solo tryptophan of the protein and the bound isoimperatorin was calculated by Förster theory as 2.90 ± 0.2 nm. Analysis of PSH values elucidated that isoimperatorin has induced some compactness in the tertiary structure of the enzyme. The results of synchronous flourescence on HSA-isoimperatorin system elucidated that the drug binds to the hydrophobic cavity (site I) on subdomain IIA that was confirmed by quenching experiments, competitive binding experiments, and docking technique.

## Figures and Tables

**Table 1:** Stern-Volmer quenching constant (*K*_SV_), quenching rate constant (*k*_*q*_), association constant (*K*_*b*_), and number of binding sites (*n*) for binding of isoimperatorin to HSA in the different temperatures.

*T* (K)	10^−4^ × *K*_SV_ (M^−1^)	10^−12^ × *K*_*q*_ (M^−1^·s^−1^)	*R* ^a^	10^−5^ × *K*_*b*_ (M^−1^)	Number of biding sites (*n*)	*R* ^a^
298	16.98 ± 0.13	16.98 ± 0.13	0.9962	11.02 ± 0.10	0.862	0.9841
303	20.19 ± 0.17	20.19 ± 0.17	0.9955	11.49 ± 0.13	0.851	0.9886
308	23.33 ± 0.16	23.33 ± 0.16	0.9965	12.71 ± 0.22	0.844	0.9950
313	26.39 ± 0.19	26.39 ± 0.19	0.9981	13.22 ± 0.17	0.822	0.9917

Data are expressed as mean ± SD of three measurements.

^a^ *R* is the correlation coefficient for the *K*_SV_ values.

**Table 2:** Thermodynamic parameters for binding isoimperatorin to HSA.

*T* (K)	Δ*S*° (J·mol^−1^·K^−1^)	Δ*H*° (kJ·mol^−1^)	Δ*G*° (kJ·mol^−1^)
298			−29.69 ± 0.09
303	178.20 ± 0.11	29.93 ± 0.13	−30.71 ± 0.07
308			−31.69 ± 0.10
313			−32.99 ± 0.08

**Table 3:** Surface hydrophobicity parameters for HSA in the absence and the presence of isoimperatorin.

Parameters	HSA	HSA-IIM complex
*K*_d_^app^ (*μ*M)	0.9 ± 0.05*	1.4 ± 0.07*
*F*_max⁡_/[HSA]	1357.14 ± 8.5**	1128.53 ± 6.3**
Surface hydrophobicity index (*F*_max⁡_/[HSA] *K*_d_^app^)	1507.94 ± 11.46**	806.12 ± 0.07**

Data are expressed as mean ± SD of three measurements. *P value < 0.0006, **P value < 0.0001.

**Table 4:** Binding constants for isoimperatorin-HSA interaction (at 295 K) in the absence and presence of the site marker.

	*K*_*b*_ × 10^−5^ (M^−1^)	*R* ^a^
HSA + isoimperatorin (in the absence of ibuprofen and warfarin)	11.10 ± 0.4*	0.9947
HSA + isoimperatorin + ibuprofen	10.99 ± 0.6*	0.9904
HSA + isoimperatorin + warfarin	7.89 ± 0.3*	0.9881

Data are expressed as mean ± SD of three measurements.

^a^ *R* is the correlation coefficient for the *K*_*b*_ values. *P value < 0.0002.

## References

[1] Hu Y-J, Liu Y, Sun T-Q, Bai A-M, Lü J-Q, Pi Z-B (2006). Binding of anti-inflammatory drug cromolyn sodium to bovine serum albumin. *International Journal of Biological Macromolecules*.

[2] Seedher N, Bhatia S (2006). Reversible binding of celecoxib and valdecoxib with human serum albumin using fluorescence spectroscopic technique. *Pharmacological Research*.

[3] Isogai H, Hirayama N (2013). In silico prediction of interactions between site II on human serum albumin and profen drugs. *ISRN Pharma*.

[4] Wang Y, Yu H, Shi X, Luo Z, Lin D, Huang M (2013). Structural mechanism of ring opening reaction of glucose by human serum albumin. *The Journal of Biological Chemistry*.

[5] Keshavarz F, Alavianmehr MM, Yousefi R (2013). Molecular interaction of benzalkonium Ibuprofenate and its discrete ingredients with human serum albumin. *Physical Chemistry Research*.

[6] Maiti TK, Ghosh KS, Debnath J, Dasgupta S (2006). Binding of all-trans retinoic acid to human serum albumin: fluorescence, FT-IR and circular dichroism studies. *International Journal of Biological Macromolecules*.

[7] Xie M-X, Long M, Liu Y, Qin C, Wang Y-D (2006). Characterization of the interaction between human serum albumin and morin. *Biochimica et Biophysica Acta*.

[8] Kanakis CD, Tarantilis PA, Polissiou MG, Diamantoglou S, Tajmir-Riahi HA (2006). Antioxidant flavonoids bind human serum albumin. *Journal of Molecular Structure*.

[9] Marumoto S, Miyazawa M (2012). Structure-activity relationships for naturally occurring coumarins as *β*-secretase inhibitor. *Bioorganic and Medicinal Chemistry*.

[10] Marumoto S, Miyazawa M (2010). Biotransformation of isoimperatorin and imperatorin by Glomerella cingulata and *β*-secretase inhibitory activity. *Bioorganic and Medicinal Chemistry*.

[11] Sajjadi SE, Shokoohinia Y, Hemmati S (2012). Isolation and identification of furanocoumarins and a phenylpropanoid from the acetone extract and identification of volatile constituents from the essential oil of *Peucedanum pastinacifolium*. *Chemistry of Natural Compounds*.

[12] Yang XB, Hou J, Liu D (2013). Biotransformation of isoimperatorin by *Cunninghamella blakesleana* AS 3.970. *Journal of Molecular Catalysis B*.

[13] Lili W, Yehong S, Qi S (2013). In vitro permeability analysis, pharmacokinetic and brain distribution study in mice of imperatorin, isoimperatorin and cnidilin in Radix *Angelicae Dahuricae*. *Fitoterapia*.

[14] Wang S, Chen Q, He L (2007). Development and validation of a gas chromatography-mass spectrometry method for the determination of isoimperatorin in rat plasma and tissue: application to the pharmacokinetic and tissue distribution study. *Journal of Chromatography B*.

[15] Sadraei H, Shokoohinia Y, Sajjadi SE, Mozafari M (2013). Antispasmodic effects of *Prangos ferulacea* acetone extract and its maincomponent osthole on ileum contraction. *Research in Pharmaceutical Sciences*.

[16] Sadraei H, Shokoohinia Y, Sajjadi SE, Ghadirian B (2012). Antispasmodic effect of osthole and *Prangos ferulacea* extract on rat uterus smooth muscle motility. *Research in Pharmaceutical Sciences*.

[17] Sajjadi S, Shokoohinia Y, Gholamzadeh S, Behbahani M, Fattahi A (2012). Antiviral evaluation of coumarins from *Prangos ferulacea* L. (Lindl). *Research in Pharmaceutical Sciences*.

[18] Sajjadi SE, Shokoohinia Y, Gholamzadeh S (2011). Chemical composition of the essential oil of the root of *Prangos ferulacea* (L.) Lindl. *Chemija*.

[19] Bijari N, Shokoohinia Y, Ashrafi-Kooshk M-R (2013). Spectroscopic study of interaction between osthole and human serum albumin: identification of possible binding site of the compound. *Journal of Luminescence*.

[20] Shi X, Liu M, Zhang M (2013). Identification of in vitro and in vivo metabolites of isoimperatorin using liquid chromatography/mass spectrometry. *Food Chemistry*.

[21] Chatterjee T, Pal A, Dey S, Chatterjee B-K, Chakrabarti P (2012). Interaction of virstatin with human serum albumin: spectroscopic analysis and molecular modeling. *Plos One*.

[22] Chen Y-M, Guo L-H (2009). Combined fluorescence and electrochemical investigation on the binding interaction between organic acid and human serum albumin. *Journal of Environmental Sciences*.

[23] Lakowicz JR (1999). *Principles of Fluorescence Spectroscopy*.

[24] Ding F, Li N, Han B, Liu F, Zhang L, Sun Y (2009). The binding of C.I. Acid Red 2 to human serum albumin: determination of binding mechanism and binding site using fluorescence spectroscopy. *Dyes and Pigments*.

[25] Ashrafi Kooshk M-R, Mansouri K, Nadi M, Khodarahmi S, Khodarahmi R (1981). In vitro anti-cancer activity of native curcumin and protein-curcumin. *Systems*.

[26] Ross PD, Subramanian S (1981). Thermodynamics of protein association reactions: forces contributing to stability. *Biochemistry*.

[27] Liu J, Tian J-N, Zhang J, Hu Z, Chen X (2003). Interaction of magnolol with bovine serum albumin: a fluorescence-quenching study. *Analytical and Bioanalytical Chemistry*.

[28] Coi A, Bianucci AM, Bonomi F, Rasmussen P, Mura GM, Ganadu ML (2008). Structural perturbation of *α*B-crystallin by zinc and temperature related to its chaperone-like activity. *International Journal of Biological Macromolecules*.

[29] Möller M, Denicola A (2002). Study of protein-ligand binding by fluorescence. *Biochemistry and Molecular Biology Education*.

[30] Khodarahmi R, Karimi SA, Ashrafi Kooshk MR, Ghadami SA, Ghobadi S, Amani M (2012). Comparative spectroscopic studies on drug binding characteristics and protein surface hydrophobicity of native and modified forms of bovine serum albumin: possible relevance to change in protein structure/function upon non-enzymatic glycation. *Spectrochimica Acta A*.

[31] Ma S-F, Anraku M, Iwao Y (2005). Hydrolysis of angiotensin II receptor blocker prodrug olmesartan medoxomil by human serum albumin and identification of its catalytic active sites. *Drug Metabolism and Disposition*.

[32] Berman HM, Westbrook J, Feng Z (2000). The protein data bank. *Nucleic Acids Research*.

[33] Sugio S, Kashima A, Mochizuki S, Noda M, Kobayashi K (1999). Crystal structure of human serum albumin at 2.5 Å resolution. *Protein Engineering*.

[34] Morris GM, Goodsell DS, Huey R, Olson AJ (1996). Distributed automated docking of flexible ligands to proteins: parallel applications of AutoDock 2.4. *Journal of Computer-Aided Molecular Design*.

[35] Faridbod F, Ganjali MR, Larijani B (2011). Interaction study of pioglitazone with albumin by fluorescence spectroscopy and molecular docking. *Spectrochimica Acta A*.

[36] Goodsell DS, Olson AJ (1990). Automated docking of substrates to proteins by simulated annealing. *Proteins*.

[37] Goodsell DS, Morris GM, Olson AJ (1996). Automated docking of flexible ligands: applications of AutoDock. *Journal of Molecular Recognition*.

[38] Sudlow G, Birkett DJ, Wade DN (1975). The characterization of two specific drug binding sites on human serum albumin. *Molecular Pharmacology*.

[39] Yue Y, Chen X, Qin J, Yao X (2009). Characterization of the mangiferin-human serum albumin complex by spectroscopic and molecular modeling approaches. *Journal of Pharmaceutical and Biomedical Analysis*.

[40] Shaw AK, Pal SK (2008). Spectroscopic studies on the effect of temperature on pH-induced folded states of human serum albumin. *Journal of Photochemistry and Photobiology B*.

[41] Ranjbar S, Ghobadi S, Khodarahmi R, Nemati H (2012). Spectroscopic characterization of furosemide binding to human carbonic anhydrase II. *International Journal of Biological Macromolecules*.

[42] Hou HN, Oi ZP, Yung YWO, Liao FL, Zhang Y, Liy Y (2008). Studies on interaction between vitamin B12 and human serum albumin. *Journal of Pharmaceutical and Biomedical Analysis*.

[43] Kalanur SS, Seetharamappa J, Kalalbandi VKA (2010). Characterization of interaction and the effect of carbamazepine on the structure of human serum albumin. *Journal of Pharmaceutical and Biomedical Analysis*.

[44] Stryer L (1968). Fluorescence spectroscopy of proteins. *Science*.

[45] Valeur B, Brochon JC (1999). *New Trends in Fluorescence Spectroscopy*.

[46] Suryawanshi V, Anbhule P, Gore A, Patil Sh, And Kolekar G (2013). A spectral deciphering the perturbation of model transporter protein (HSA) by antibacterial pyrimidine derivative: pharmacokinetic and biophysical insights. *Journal of Photochemistry and Photobiology B*.

[47] Yuan T, Weljie AM, Vogel HJ (1998). Tryptophan fluorescence quenching by methionine and selenomethionine residues of calmodulin: orientation of peptide and protein binding. *Biochemistry*.

[48] O’Haver TC, Fell AF, Smith G (1982). Derivative spectroscopy and its applications in analysis. *Analytical Proceedings*.

[49] Wang Y-Q, Tang B-P, Zhang H-M, Zhou Q-H, Zhang G-C (2009). Studies on the interaction between imidacloprid and human serum albumin: spectroscopic approach. *Journal of Photochemistry and Photobiology B*.

[50] Kim S-J, Rhee H-W, Park H-Y, Kim H-S, Hong J-I (2013). Fluorescent probes designed for detecting human serum albumin on the basis of its pseudo-esterase activity. *Bioorganic & Medicinal Chemistry Letters*.

[51] Ascenzi P, Gioia M, Fanali G, Coletta M, Fasano M (2012). Pseudo-enzymatic hydrolysis of 4-nitrophenyl acetate by human serum albumin: pH-dependence of rates of individual steps. *Biochemical and Biophysical Research Communications*.

